# Curcumin's effects on the reproductive and nervous systems

**DOI:** 10.17179/excli2017-338

**Published:** 2017-05-15

**Authors:** Ali Noorafshan, Ali Rafati, Saied Karbalay-Doust

**Affiliations:** 1 Histomorphometry and Stereology Research Center, Shiraz University of Medical Sciences, Shiraz, Iran; 2 Anatomy Department, School of Medicine, Shiraz University of Medical Sciences, Shiraz, Iran; 3 Department of Physiology, Shiraz University of Medical Sciences, Shiraz, Iran

## ⁯⁯

Dear Editor,

Curcumin extracted from the **Curcuma longa** rhizome is the major constituent of turmeric and used as a spice in food preparation. Since this substance is available everywhere and could be obtained with a reasonable price, we decided to conduct various researches on it thoroughly. It was considered as a research topic/priority in Histomorphometry and Stereology Research Center of Shiraz University of Medical Sciences as it could be recommended to public after passing the biological examinations in animal and human models. Therefore, evaluation of protective effects of curcumin began in 2010 in our research centre. In the first studies, different dosages of curcumin were assessed to find the safe doses of it. This survey showed that 100 mg/kg/day was an optimum dose in rats (Kheradpezhouh et al., 2010[3]). The reproductive and nervous systems health are of importance in the University and have been given priorities. Therefore, in the next steps of the research, we focused on the protective properties of curcumin on the male reproductive system in the experimental toxicity in animal models (Noorafshan et al., 2010[12], 2011[11]; Noorafshan and Karbalay-Doust, 2012[10]; Karbalay-Doust and Noorafshan, 2011[1]). The research on curcumin was followed by evaluation of curcumin's effects on peripheral and central nervous systems. The peripheral nerve injuries (sciatic nerve) were induced in rat models and co-treatment of curcumin in these conditions assessed (Noorafshan et al., 2011[13][14]). Since emotional stress are the indispensable parts of life, the next evaluations were arranged to show whether curcumin could improve behavioural and structural changes of the brain after induction of stress and depression models (Noorafshan et al., 2013[6], 2014[4], 2015[7][5]). The preservative agents and artificial dyes are used commonly in food and drug industries, and their adverse impacts on the mammalian bodies have been approved. Nevertheless, finding shielding agents that could be used after consuming these substances has received less attention. Therefore, curcumin's effects were assessed in the brain tissue after exposure to preservative agents and artificial dyes (Karimfar et al., 2014[2]; Noorafshan et al., 2013[15], 2015[8]). Publishing of these articles draw attention of the journal entitled Current Pharmaceutical Design. We were asked to write a review for them in 2013 and up to now it has been cited by many authors (Noorafshan and Ashkani-Esfahani, 2013[9]). In above-mentioned research, curcumin was able to protect the tissues even though no obvious adverse effects were seen except for the formation of renal and bladder stone (unreported report).

In continuation of the above-mentioned articles, it is our honour to present our works in EXCLI Journal. 

## Acknowledgements

This work was supported by grant No. 96-01-14 from the Histomorphometry and Stereology Research Center of Shiraz University of Medical Sciences.

## Conflict of interest

The authors declare no conflict of interest. 
